# SSTrack: An Automatic Sunspot Identification and Tracking Algorithm to Support the Measurement of Sunspot Rotation

**DOI:** 10.1007/s11207-026-02689-z

**Published:** 2026-06-12

**Authors:** Charlotte Proverbs, Daniel Brown

**Affiliations:** Jeremiah Horrocks Institute, University of Lancashire, Preston, PR1 2HE UK

**Keywords:** Sunspots, Rotation, Solar Dynamics Observatory

## Abstract

Motions of sunspots cause the coronal magnetic field to become deformed with the effect that energy can be stored in the coronal magnetic field. This energy can be released through events such as solar flares. Sunspots are known to rotate about their umbral centres, and this rotation contributes to the build-up of energy within an active region that can be released during an eruptive solar event. To understand the relationship between rotational forms of sunspot dynamics and solar activity, a large, unbiased statistical survey of sunspots is desirable.

To generate such a statistical sample, a fully automatic sunspot identification and tracking method, SSTrack, is developed. This method is tested on a previously analysed four-month sample of active regions generated using a semi-automatic sunspot rotation tool. The new method is designed to be applied to long periods of observations so that large samples can be efficiently obtained, while working at a high-cadence to effectively capture fine-scale-behaviour, such as sunspot splitting and mergers. SSTrack is able to identify fifty-four of the fifty-six sunspots in the four-month sample as well as an additional forty-three sunspots not found by the semi-automatic method. It is able to identify many of the fifty-four commonly found sunspots earlier and track them for longer than the semi-automatic method. The rotation about umbral centres is calculated for each sunspot using the tracking data from both methods, and when considering only overlapping observations per sunspot the methods show good agreement. The semi-automatic method is found to detect greater rotation when a sunspot undergoes fragmentation due to the changing structure overly influencing the determination of the centre of the sunspot.

## Introduction

The Sun’s magnetic field protrudes from the solar interior, where it is generated, into the solar atmosphere where it can manifest as darker and cooler regions on the photosphere known as sunspots. Sunspot dynamics are intrinsically linked to variations in the atmospheric magnetic field and have the ability to inject energy into active regions during the lead up to eruptive events, such as coronal mass ejections and solar flares (Kumar et al. 2013; Vemareddy, Cheng, and Ravindra 2016; Vemareddy, Ambastha, and Maurya 2012). These dynamics can include sunspot rotation (Brown and Walker 2021), pair interactions (Ravindra, Yoshimura, and Dasso 2011; Yan et al. 2015), shear (Kazachenko et al. 2009, 2010; Vemareddy et al. 2012) and structural changes including splitting and merging (Louis et al. 2012, 2014).

Sunspots have been observed to rotate about their umbral centres at rates of around one degree per hour for several days, building up energy in active regions that can lead to an eruption or a solar flare (Sturrock et al. 2015; Brown et al. 2003; Zhang, Liu, and Zhang 2008). The relationship between sunspot rotation and different types of solar activity has been investigated, with relationships identified across limited case studies of 1 – 10 rotating sunspots, such as Li and Liu (2015), Min and Chae (2009), Vemareddy, Ambastha, and Maurya (2012), Zhang, Liu, and Zhang (2008), Török et al. (2013), Yan et al. (2012), Kazachenko et al. (2009), Georgoulis and LaBonte (2006), Gopasyuk and Gopasyuk (2005), Gopasyuk and Kosovichev (2011), Jiang et al. (2012), Li et al. (2010), Régnier and Canfield (2006), Ruan et al. (2014), Srivastava et al. (2010), Tian, Alexander, and Nightingale (2008), Wang et al. (2014, 2016), Yan and Qu (2007), Yan et al. (2009, 2018), Zheng et al. (2017), Denker et al. (2025) and Grimes, Pintér, and Morgan (2020). Many of these studies found that solar flares were most likely to occur as the sunspot rotation rate reached its maximum, implying that the latter could be the source of the build-up of magnetic energy in the corona itself, caused by the large-scale twisting of magnetic flux tubes (Yan, Qu, and Kong 2008). Sunspot rotation has also been linked to coronal mass ejections through individual case studies of CMEs and a related active region, for example Yan et al. (2012), Török et al. (2013), Yan et al. (2018).

In order to conduct a large scale statistical analysis of sunspot dynamics, such as sunspot rotation, a large sample of sunspots that are tracked for their durations as they transit the solar disk need to be produced. Generating such a sample manually (such as those compiled by Walker (2018)) requires time-consuming and labour intensive analysis of large quantities of photospheric observations. This is prone to classification errors on the part of the observer.

Multiple automated methods to track solar regions have been developed. AutoTAB (Automatic Tracking Algorithm for Bipolar Magnetic Regions) (Sreedevi et al. 2023) generated a catalogue of automatically tracked bipolar magnetic regions using line-of-sight magnetograms using data from MDI and SDO/HMI from 1996 – 2019. Although this catalogue is expansive, the temporal cadence of the HMI data is reduced to one image every 96 minutes and the data is re-binned from the original $4096\times 4096$ pixels to $1024 \times 1024$ to match the resolution and available cadence of the MDI data and to also reduce computational time. Re-binning the data to this extent means that fine structures in bipolar magnetic regions will not be detected, and a cadence of 96 minutes is not sufficient to monitor the splitting and merging of sunspots. Similarly, STARA (Sunspot Tracking and Recognition Algorithm) from Watson et al. (2009) used MDI white-light images from 1997 – 2003 to generate a sunspot catalogue, however these images were sampled at a 6-hour cadence, which does not allow for the tracking of sunspot dynamics such as sunspot rotation and shear. The STARA method has also been adapted for use with SDO/HMI data and other ground-based observatories, with one measurement of a sunspot umbrae being made per day (Watson, Penn, and Livingston 2014). Finally, the HMIDD (SDO/HMI – Debrecen Sunspot Data) from Baranyi, Győri, and Ludmány (2016) automatically generated a sunspot catalogue using SDO/HMI continuum data at full resolution, but at an hourly cadence.

Other approaches, such as DAVE4VM (Differential Affine Velocity Estimator for Vector Magnetograms) by Schuck (2008) and the MLT (Multi-Layered Thresholding) method by Grimes, Pintér, and Morgan (2020), are capable of using full-resolution data from SDO/HMI at a very high cadence to calculate detailed measurements of sunspots and magnetic regions. However, these methods are computationally expensive, and so are not suitable for generating large catalogues of sunspot data.

In order to statistically analyse sunspot properties we need a catalogue of sunspots that cover long series of data at full resolution with a small temporal cadence. Current efforts either require manual involvement or are computationally expensive, which reduces the size of the data series that can be analysed, or utilise automatic methods, but restrict the resolution or temporal cadence to reduce the computational time required.

The purpose of this paper is twofold. First, a fully automatic method to identify and track sunspots is developed (SSTrack). The aim is to develop a method that can be tractably run on sequences of months-years of observations at a cadence of a few minutes in order to capture fine-scale evolution and to produce location information suitable for passing to sunspot rotation algorithms. SSTrack identifies sunspots in photospheric continuum data from the Heliospheric and Magnetic Imager (HMI) instrument (Schou et al. 2012) on NASA’s Solar Dynamics Observatory (SDO) (Pesnell, Thompson, and Chamberlin 2012). Identified sunspots are then tracked across sequences of observations to determine the locations of the sunspots (along with other key properties such as umbral area) as they transit the solar disk to produce a catalogue of sunspot locations and properties. The method is able to identify when sunspots are undergoing structural changes, such as merging and splitting, and is able to separate these more permanent changes from flickering light bridges.

Secondly, the SSTrack method is tested on a four-month series of data covering 1 May 2013 – 31 August 2013 that has been previously analysed using a by-eye method (Walker 2018) to assess the performance of the fully-automatic method. This semi-automated method (Brown and Walker 2021) requires some initial identification about the sunspot to be tracked as well as the list of the observations in which to track it, however, the list of observations must be determined manually as existence of the sunspot is assumed for the duration of the full list and a robust approach to ensure continuity of detection is taken, which can be indiscriminate of finer sunspot behaviour such as sunspot splitting and mergers. The method developed here automatically determines the duration of tracking of a sunspot (within the available observations) and takes a more elegant approach to identifying sunspot splitting and merger.

## Method

The sunspot identification and tracking method (SSTrack) comprises three separate processes:

- sunspot identification in individual observations,
- linking sunspots between frames,
- sunspot sub-umbral structure determination.

The identification algorithm takes a list of SDO/HMI FITS (‘hmi.Ic_45s’) files at a three-minute cadence as input and produces as output a set of metadata files (one per observation) containing the different sunspots identified in the observation along with key characteristics such as weighted centre-of-mass, area, radius and associated uncertainties. Stray light corrected data for SDO/HMI is available at a cadence of one image per day (Norton et al. 2026), with more available on request, however to generate a suitable statistical sample we choose to work on the most voluminous catalogue. In addition to lone sunspots, the algorithm attempts to identify sunspot umbra that sit within a shared penumbra, this may identify sunspots in the process of merging or splitting and may flag some umbrae as being in a ‘grey zone’ where they may or may not be discrete sunspots. For this step, the order that observations are processed is not important allowing for the use of parallel processing.

The linking algorithm uses the metadata files generated by the identification algorithm to connect sunspots between successive observations so their location can be tracked over the entire observation period. Each sunspot is assigned to its corresponding active region.

The structure determination stage looks at any sunspot flagged in the grey zone in the context of its surrounding observations to determine whether a sunspot is fragmenting into two spots or two sunspots are merging into one (or it is just a spurious identification). It also applies a second linking process to join any final sunspot strands that have previously been missed.

The following sections discuss these stages in detail.

### Sunspot Identification Method

**Figure 1:**
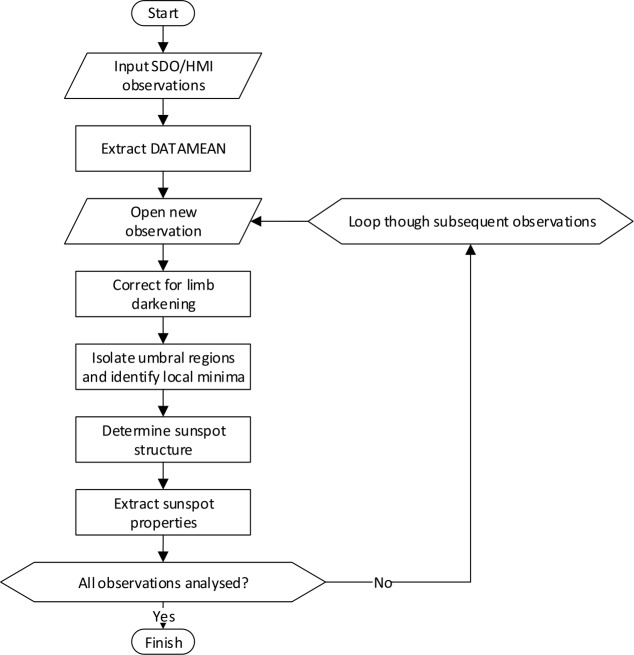
Method flow chart of the sunspot identification algorithm.

The sunspot identification method is divided into smaller stages, as shown by Figure 1. The data is first corrected for limb darkening effects, and the umbral regions of the sunspots are identified. The structure of the sunspots are determined and properties are extracted and recorded per observation.

#### Data Preparation

As a sunspot crosses the solar disk, the observed intensity of the umbral and penumbral regions vary due to limb darkening effects. To define an umbral pixel intensity threshold, this effect must be corrected for. The limb darkening correction used for this work is discussed in detail in Brown and Walker (2021). The observations are corrected using

1 $$ I_{corr}(x,y) = \frac{I(x,y)}{\sum _{i=0}^{4} a_{i} \cos ^{i} \theta} $$

where $(x,y)$ is the location of the pixel being corrected relative to disk centre, and $\cos{\theta}$ is given by

2 $$ \cos \theta = \sqrt{1 - \frac{\left (x^{2} + y^{2} \right )}{r_{s}^{2}}} $$

where $r_{s}$ is the radius of the Sun, also in pixels. The values of $a_{i}$ are given by Brown and Walker (2021) as $a_{0}=0.278$, $a_{1}=1.673$, $a_{2}=-2.336$, $a_{3}=2.133$, and $a_{4}=-0.748$.

Once the limb darkening effects are corrected for, a fixed pixel intensity threshold is set for the umbral region of the sunspot. Determination of this threshold follows Brown and Walker (2021), the datamean value is extracted from the header of all of the observations under analysis and the mean of those values is calculated using

3 $$ \bar{I} = \frac{1}{N} \sum ^{N}_{i=1}\textsc{DATAMEAN}_{i} $$

The umbral region of the sunspot is defined as

4 $$ I_{pix} \leq 0.6 \bar{I} $$

and the penumbral region of the sunspot is defined by

5 $$ 0.6\bar{I}< I_{pix} \leq 1.05 \bar{I} $$

**Figure 2:**
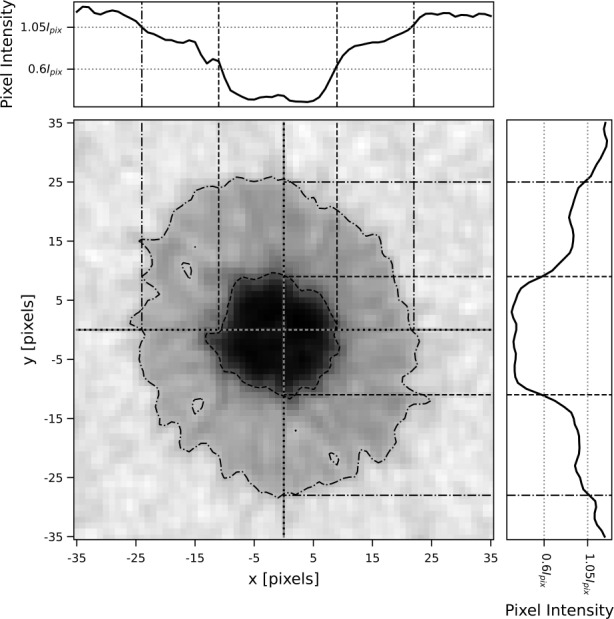
A reprojected SDO/HMI image with the horizontal and vertical intensity profiles across the centre of the sunspot. The umbral and penumbral thresholds are plotted on the sunspot image and the intensity profiles.

These levels and regions are illustrated in Figure 2.

#### Sunspot Identification

Within a given observation, all pixels that have an intensity below the umbral intensity threshold (as defined by Equation 4) are flagged as umbral pixels. These are then divided into groups of connected umbral pixels (such that any two pixels in the group can be traced through a set of horizontally and vertically adjacent umbral pixels). Each one of these groups represents either a sunspot, or a group of sunspots in a shared penumbra.

**Figure 3:**
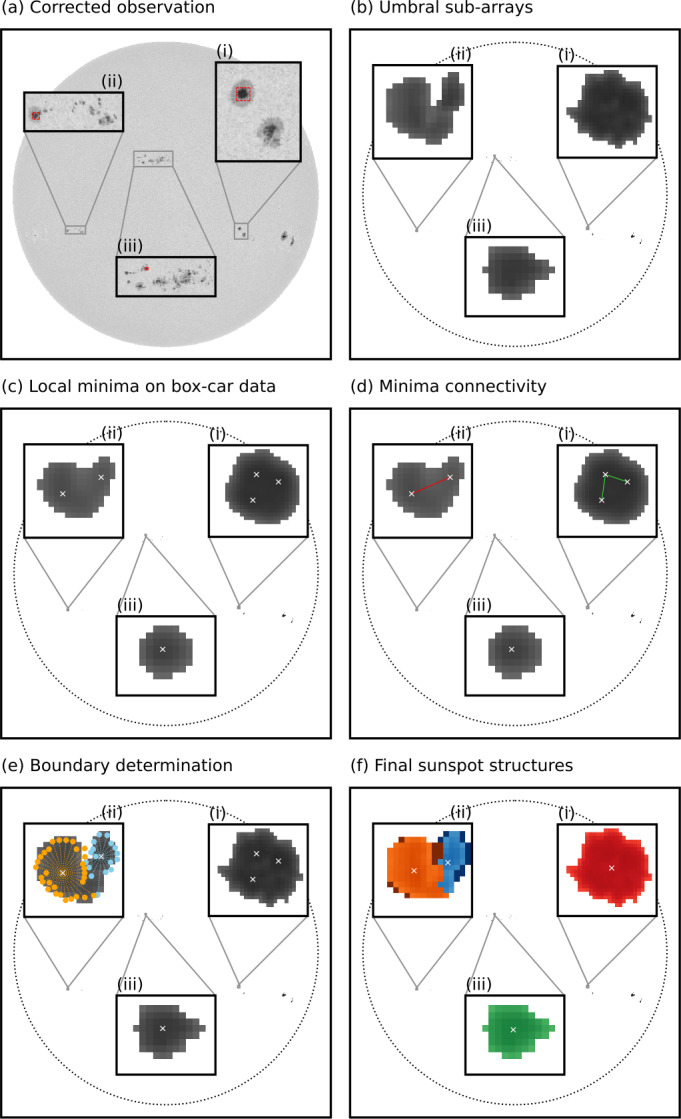
Sunspot structure determination. (a) The limb corrected SDO/HMI observation, the sub-arrays contain active regions (i) 11734, (ii) 11730 and (iii) 11731. (b) The sub-arrays containing the islands of umbral pixels. (c) The local minima location within the filtered sub-arrays. (d) The links between the local minima. (e) The boundaries of the sub-structure within the sub-arrays. (f) The final sunspot structure.

A $5\times 5$ box-car filter is applied to the data and local minima within any given group are found. The filter removes excess noise and ensures that identified local minima are minima of the signal rather than the noise. These represent the minima of each potential sunspot within the group. If a single local minima is identified, then the connected group is taken to be a single sunspot. This scenario is presented as case (iii) in Figure 3.

**Figure 4:**
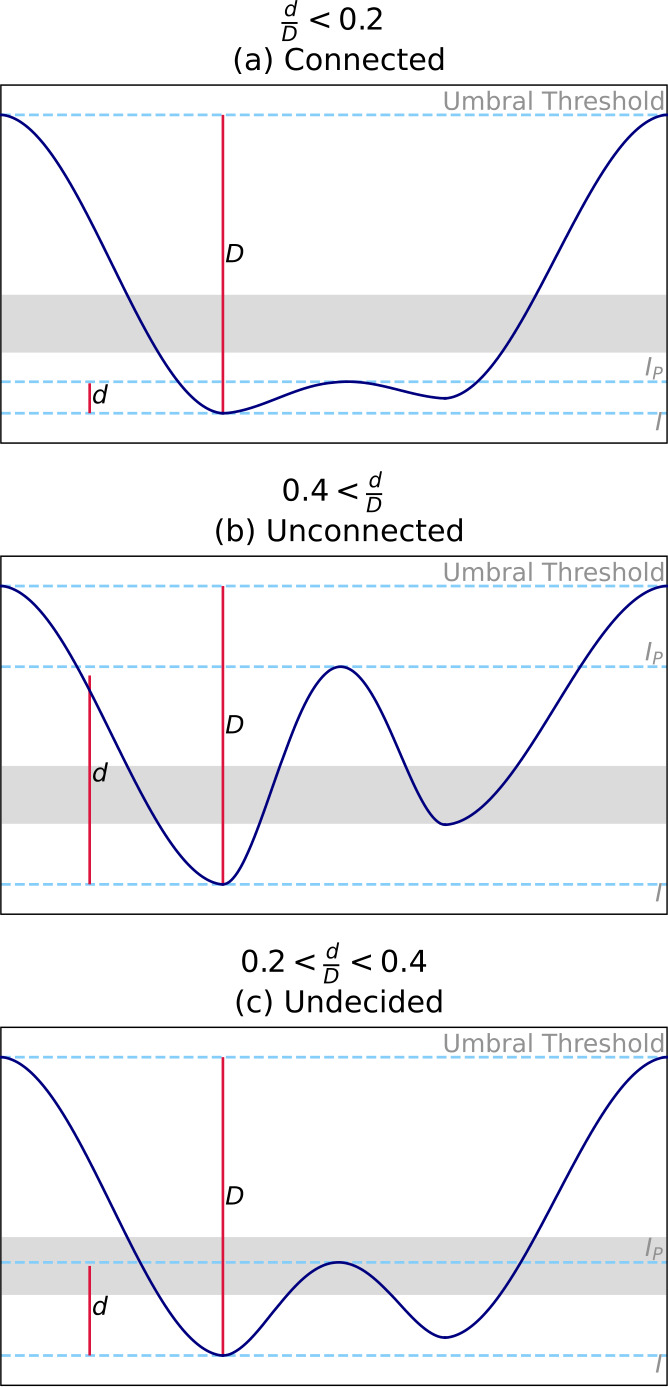
The navy line represents the interpolated intensity between two minima in a sunspot region. $I$ is the intensity at the smallest minima, $I_{P}$ is the intensity at the maximum value between the minima and $D$ and $d$ are the differences between $I$ and the DATAMEAN threshold and $I_{P}$, respectively. If the peak between the minima does not reach the grey region the minima are considered to be connected (a). If the peak between the minima surpasses the grey region the minima are considered to be unconnected (b). If $I_{P}$ lands within the grey region (c) the minima are recorded as being temporarily connected but at risk of separating in future frames.

If there are multiple minima within a group then a further process is followed. The local minima are sorted by ascending intensity. The local minima with the smallest intensity is considered to be part of a sunspot component. All the other minima are compared (in order of ascending local minima intensity) to the smallest local minima to determine whether they are connected or not (i.e. part of the same sunspot). To determine if the minima are connected, a line of intensity is interpolated between the two minima. The minima are considered to be connected if

6 $$ \frac{d}{D} \le 0.2 $$

where $d$ is the difference between the intensity peak of the interpolated line and the smallest intensity minima, and $D$ is the difference between the umbral intensity threshold ($0.6 \bar{I}$) and the smallest intensity minima. If the minima are determined to be connected, the interpolated line would take a form similar to that of Figure 4a, then the second minima is added to the first to form a single sunspot. This scenario is presented in Figure 3, case (i). Here there are three local minima within the umbral region that are part of the same sunspot structure. The minima are considered to be unconnected and belong to two separate sunspots within the same umbral region if

7 $$ \frac{d}{D} > 0.4 $$

An example of this is shown in Figure 3 case (ii), and the interpolated intensity profile between the two minima is similar to that of Figure 4b.

If the minima comparison is

8 $$ 0.2 < \frac{d}{D} \le 0.4 $$

the structure of the umbral region is classed as undecided and a final decision is made once the behaviour of the sunspot can be placed in the context of the surrounding linked observations (see Section 2.3. Generally the sunspots that fall in this category are those that are either splitting or merging in the surrounding frames. Examples of these relations are represented in Figure 4c.

This *grey zone* is designed to build in some memory, or hysteresis, to the algorithm so that in the case of e.g., two sunspots that are merging, the algorithm continues to identify them as separate until the merger is complete, and avoids flickering between identifying one or two sunspot in subsequent observations due to random noise.

This process is repeated between every minima in the sub-array to determine which minima are connected and how many different sunspots are within the umbral region. If all of the minima within the umbral region are considered to be part of the same sunspot, all of the pixels within the umbral region are assigned to the single sunspot. If there are multiple sunspots within one umbral region, the boundary between the sunspots must be identified. For each sunspot, an estimate for the typical radius of the sunspot is calculated by interpolating a line radially outwards from each local minima at 90° increments until a turning point or the umbral intensity threshold is reached. The radial estimate is the average of these radii,

9 $$ \hat{r}=\frac{1}{4}\sum _{i=0}^{4} r_{i} $$

The inverse of this estimated radius defines the angular increment used for the boundary definition

10 $$\hat{r}\delta \theta =1\;⟹\;\delta \theta =\frac{1}{\hat{r}}$$

Using this definition, one increment change in the angle around the local minima is approximately equivalent to moving no more than one pixel along the circumference of the umbral region. A line is then interpolated radially outwards from the local minima at increments of one pixel until either the edge of the umbral region is reached or there is a turning point in the intensity. This pixel is considered to be the boundary of the sunspot. This is repeated through 360° for each local minima within the umbral region (Figure 3e). All of the pixels within these boundaries are collected and assigned to the correct sunspot. If there are any unassigned pixels within the umbral grouping these are assigned to the closest sunspot.

The pixels assigned to each sunspot are then used to calculate the centre of mass of the sunspot, the centre of mass weighted for intensity, the maximum radius (the maximum distance in pixels from the weighted centre of mass to the edge of the umbra in the sunspot), the umbral area (the total number of pixels included in the sunspot umbra), the structure and linking criterion alongside the errors associated with these parameters. An overview of the recorded sunspot properties is provided in Appendix C. Once all sunspots are identified in a given observation, the process is then repeated for the next observation in the data set. As the order that the observations are analysed does not impact the results for the sunspot identification process, this step can be carried out in a parallel manner.

### Sunspot Linking Method

Once all of the sunspots across the sequence of observations are identified they are linked between frames to allow the tracking of sunspots. All of the sunspots in the first frame are assigned integer numbers from $1-n$ where $n$ is the number of sunspots in the frame. All observations in the subsequent frame are compared to those in the previous observation to determine if the sunspots are linked to any previous sunspot or if they are new emerging sunspots.

Suppose the current observation under analysis is frame $t$, with a sunspot’s weighted centre of mass $(x_{t}, y_{t})$ and the previous observation is frame $t-1$, with a sunspot’s weighted centre of mass $(x_{t-1}, y_{t-1})$. The effect of the Sun’s differential rotation between frame $t$ and frame $t-1$ is removed from observation $t$, these sunspots are then referred to as, $(\bar{x}_{t}, \bar{y}_{t})$. The distances between the sunspot in frame $\bar{t}$ and any sunspot from the previous frame $t-1$ is given by

11 $$ d = \sqrt{\left ( \bar{x}_{t} - x_{t-1} \right )^{2} + \left ( \bar{y}_{t} - y_{t-1}\right )^{2}} $$

In order for two sunspots to be considered as linked, the distance $d$ between them is required to be less that the mean radius of the sunspots in the frames under comparison

12 $$ r_{crit} = \frac{1}{2}\left ( r_{t} + r_{t-1} \right ) $$

This ensures that sunspots can only be linked to near-neighbours in subsequent frames.

There are two special cases that can occur during the linking process. Firstly, multiple sunspots from frame $t$ could track back and satisfy the linking criteria for a single sunspot in frame $t-1$. These sunspots typically fall within the ‘undecided’ region (Equation 8) from the structure identification process. The sunspots are then recorded as two separate structures, to be more thoroughly checked once the structural changes are placed in the context of the surrounding observations. In this case the sunspots are considered to be potentially splitting as they were connected in the previous frame but disconnected in the current frame. The other special case occurs when a single sunspot in the current frame (frame $t$) tracks back to multiple sunspots in the previous frame (frame $t-1$). In this case, the sunspots also typically have an ‘undecided’ structure and are considered to be potentially merging in frame $t-1$ to a single sunspot in frame $t$.

If no sunspots from the previous frames fulfil this criteria, then the process is repeated between frames $t$ and $t-2$. This allows for the occasional ‘dud’ frame in the sequence. To avoid the potential ambiguity of trying to link sunspots across large time gaps, the maximum time between frames that can be compared is set to one hour. This process is repeated for all sunspots in subsequent frames in the dataset.

To assign each sunspot to an active region, data from NOAA’s daily Solar Region Summary (SRS) is downloaded for each day in the SDO/HMI FITS file list. The active region number, latitude, longitude and observation datetime is extracted from the NOAA SRS data. Every recorded sunspot observation from the SSTrack method is differentially rotated to the datetime of the NOAA SRS observation to allow the sunspot locations to be as close as possible to the recorded NOAA SRS positions. Each sunspot observation is then assigned to the closest active region latitude and longitude. Once every sunspot observation has been assigned, all observations of unique sunspots are automatically checked to ensure that the sunspot is consistently assigned to a single active region. If a sunspot is assigned to multiple active regions, the active region that it is assigned to the most takes precedence.

### Sunspot Sub-Umbral Structure Determination

The final step is to resolve the status of the sunspots that fall within the ‘grey zone’ and determine whether they are/become separate sunspots or if they are part of the same sub-umbral structure. To finalise the linking of the sunspots, each observation is placed within the context of the active region. A linear convolution filter (with window size 100) is applied to the recorded sunspot area of each sunspot, the smoothed area result is deducted from the original area to isolate the unexpected variations. Observations where the sunspot structure criteria falls within Equation 8 are extracted. Consecutive observations where sunspots fall within the ‘undecided’ region are confirmed to be splitting or merging sunspots. Non-consecutive ‘undecided’ observations where the area variation exceeded 55 pixels are considered to be part of the same structure. These temporary variations in the sunspot structure are generally caused by light-bridges forming across the umbra. Once the components are merged together the recorded values are combined and attributes such as the weighted centre of mass and area are recalculated using the new pixel allocations.

A second sunspot linking method is then applied to the data, to connect sunspots together that would not have been considered before the structure determination method. The final hour of data for sunspot A is extracted and the mean weighted centre of mass coordinates, mean radius and median reprojected area is determined for these observations.

A new area criterion is calculated for the second sunspot linking run. The area limit is defined by

13 $$ A_{\mathrm{lim}} = A_{\mathrm{area}} \pm 5\sigma $$

14 $$ \sigma =0.0592 \times A_{\mathrm{area}} + 4.6658 $$

where $\sigma $ is the area limit factor and $A_{\mathrm{area}}$ is the area of sunspot A. The derivation of this criteria is discussed in Appendix A. Sunspots A and B are considered to be connected if they fulfilled three criteria:

- The first observation of sunspot B is within ten-hours of the final observation of sunspot A
- The median area from the first hour of observations for sunspot B is within $A_{\mathrm{lim}}$
- The distance between the mean centre of mass for sunspot A and B is less than the mean radius of sunspot A.

The second linking process is the final stage of the sunspot identification and tracking method. The metadata for each active region is recorded and ready to input into sunspot dynamic methods.

## Results

In this section, the performance of the SSTrack algorithm is evaluated by analysis of the four-month sample of ‘hmi.Ic_45s’ data at a three-minute cadence, running from 1 May – 31 August 2013, previously studied by Walker (2018) which was identified and tracked using a semi-automated approach where initial identification is performed by eye but tracking is carried out automatically. The method used by Walker (2018) identifies contiguous groups of umbral pixels (as described in Section 2.1) but does not try to identify any substructure within these groups.

In addition, Walker (2018) apply size and duration criteria to the sunspots in their sample. A sunspot is included only if it maintains an area greater than $49\pi $ pixel^2^ (though it is allowed to drop to $36\pi $ pixel^2^ temporarily) for a period greater than 24 hours. Walker also insists that the sunspots are not too close to the solar limb, and only consider sunspots located within 60° of disk centre (such that $\cos{\theta}\ge 0.5$ in Equation 2). Walker (2018) were interested in sunspot rotation (about their umbral centres) and these criteria were used to ensure follow-on rotation analysis could be performed.

For comparison, the results of the SSTrack method are filtered to also meet these criteria. Some sunspots from Walker (2018) were found to not meet the above filters and were included in the sample by error (see Appendix B). These sunspots have been removed from the sample to allow a fair comparison between the methods. The rotation profiles for the active regions identified using the SSTrack method are available at 10.17030/uclan.data.00000652. An overview of the properties recorded in these files is in Appendix C.

This section will compare the following properties of the two samples; the number of sunspots per active region, the amount of time that each sunspot is observed for, the observed sunspot rotation.

### Sunspot Number Comparison

**Figure 5:**
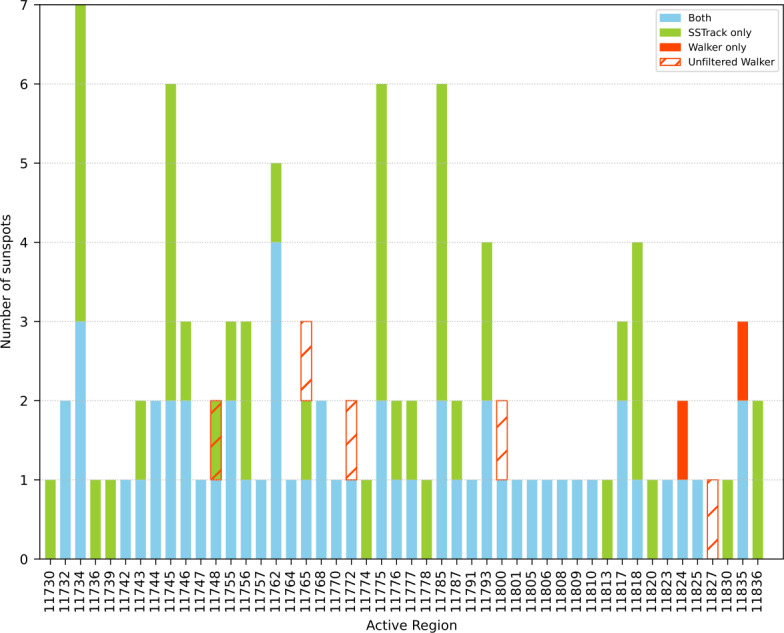
A histogram showing the number of sunspots identified per active region by the two sunspot identification methods. Sunspots identified by both methods are highlighted in blue, by only the SSTrack method in green and by only the Walker method in red. Sunspots identified by the Walker method that do not pass the area filter are shown as red hatches.

Over the four-month period, the SSTrack method identifies 97 sunspots over 47 active regions while Walker (2018) identify 56 sunspots over 38 active regions. The number of sunspots identified per method and per active region is shown in Figure 5. This shows that the SSTrack method identifies 54 of the sunspots found by Walker, the two non-identified sunspots are from AR 11824 and AR 11835. The reasons why Walker identifies more sunspots are presented in Table 1.

**Table 1:** Active regions where the Walker method was able to identify additional sunspots than the SSTrack method with explanation. Throughout this work, sunspots identified by Walker are referred to with subscript $W$, and sunspots identified using SSTrack (Proverbs and Brown) are referred to with subscript $P$.

AR	Why
11824	Walker finds sunspot $B_{W}$ to have an area exceeding the threshold for 24.6 hours, whereas sunspot $B_{P}$ remains above the threshold for only 16.95 hours so is excluded according to Walker’s selection criteria
11835	Sunspot $A_{W}$ and $A_{P}$ fragments, Walker considers the two fragmented sunspots to be two new sunspots, $AA_{W}$ and $AB_{W}$. SSTrack does not relabel the large sunspot. This active region is discussed in Section 3.1.1.

However, the SSTrack method identifies 43 sunspots that Walker (2018) miss, including nine active regions that did not have associated sunspots in the Walker dataset (active regions 11730, 11736, 11739, 11774, 11778, 11813, 11820, 11830 and 11836). Generally, the 54 communally identified sunspots show good agreement, with the SSTrack method showing an increased ability to track the structural changes (e.g., splitting and mergers) within the umbral regions of the sunspots in comparison to the Walker method. An example of an active region with good agreement is discussed in Section 3.1.2.

**Figure 6:**
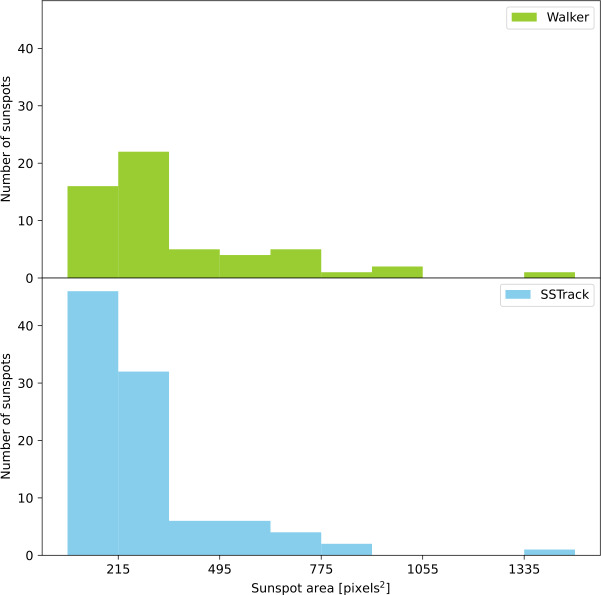
A histogram showing the mean area distribution of sunspots identified with the SSTrack method, and the Walker method.

Figure 5 also shows that the SSTrack method typically identifies more sunspots per active region. The mean area distributions of the sunspots found by each method are plotted as histograms in Figure 6. This shows that the SSTrack method has a greater sensitivity to smaller sunspots than the Walker method. There are two main reasons for this, the first is that the ‘by-eye’ identification approach of Walker may have dismissed some of the smaller sunspots that are close to the minimum area threshold but are picked up by the SSTrack method. The second is that the SSTrack method is designed to identify substructure in an umbral grouping and a single large sunspot identified by Walker (2018) may be resolved into multiple smaller sunspots by the SSTrack detection method.

Active region 11836 is included in this analysis for completeness, however this active region may have been omitted from the original Walker sample as the final observations of this active region occurs after the end of the sample period.

#### Analysis of AR11835: Walker Detects Additional Sunspots

**Figure 7:**
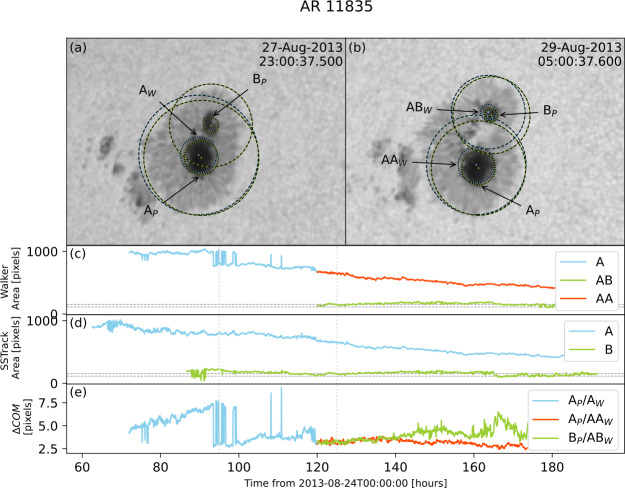
Tracking profiles for AR 11835: (**a,b**) image of the sunspots from 27 August 2013 at 23:00:37 UT and 29 August 2013 at 05:00:37 UT, respectively, with the identified penumbral and umbral radii overlaid from both identification methods; (**c**): the reprojected umbral area profile for the sunspots identified by the Walker method; (**d**): the reprojected umbral area profile for the sunspots identified by the SSTrack method; (**e**): The difference in recorded sunspot centre of mass for the comparable sunspots identified by both methods.

AR 11835 is a case where Walker (2018) identified a sunspot that the SSTrack method does not. Summary observations from the active region can be seen in Figure 7, and it can be seen from the sequence of observations that the sunspot is undergoing fragmentation.

Walker (2018) manually choose a time (around 120 hours after the epoch) before which only the single large sunspot is tracked and after which the two fragmented sunspots are tracked separately, with the two sunspots receiving new labels ($AA_{W}$ and $AB_{W}$). Flickering in the area profile (Figure 7c coinciding with the observation in panel a) suggests that the fragmentation could be occurring around 90 hours after the epoch.

The SSTrack method (by design) does not relabel the large sunspot on fragmentation, but continues to track Walker’s $AA_{W}$ as part of sunspot $A_{P}$ (Figure 7d). The smaller sunspot is identified earlier (around 85 hours after the epoch) illustrating the ability of the SSTrack method to identify and track fragmenting sunspots.

The SSTrack method has detected the additional sunspot in the Walker sample, it has just been classified differently so registers in Figure 5 as a non-detection.

The difference in the centres of sunspots in the two samples are shown in Figure 7e. There is generally good agreement in the locations of the two with the slightly larger discrepancy between 75 and 90 hours being due to the SSTrack method using a weighted centre of mass rather than the equal weighting used by Walker (2018). This leads to the pre-fragmenting spur at the top of the sunspot having enhanced weighting in the Walker calculations producing a slightly offset calculated sunspot centre.

#### Analysis of AR 11808: Good Agreement Case

**Figure 8:**
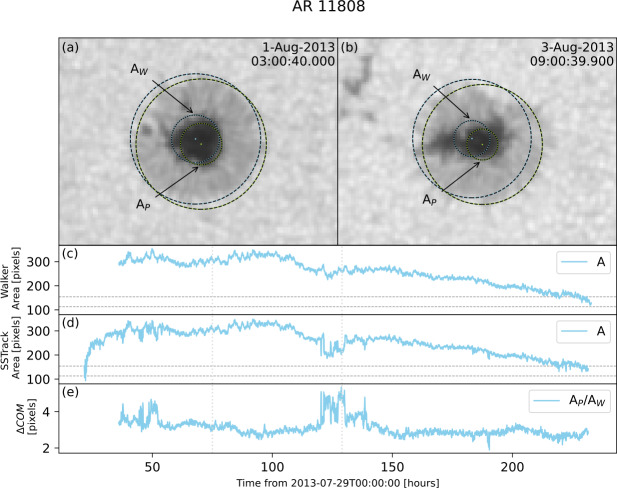
Tracking profiles for AR 11808: (**a,b**) image of the sunspots from 1 August 2013 at 03:00:40 UT and 3 August 2013 at 09:00:40 UT, respectively, with the identified penumbral and umbral radii overlaid from both identification methods; (**c**): the reprojected umbral area profile for the sunspots identified by the Walker method; (**d**): the reprojected umbral area profile for the sunspots identified by the SSTrack method; (**e**): The difference in recorded sunspot centre of mass for the comparable sunspots identified by both methods.

This example illustrates how for simple sunspot structures the two methods perform comparably. AR 11808 consists of a large, circular sunspot that elongates as it traverses the solar disk, the areas and the differences in sunspot centres are shown in Figure 8. There is some difference in centre of mass during the maximal point of elongation (Figures 8b and e), but overall the two methods show good agreement. The SSTrack method identifies the sunspot approximately 24 hours earlier than the Walker method, but the identified areas are very similar throughout the observations and the distance between centres is consistently below 5 pixels. Overall the methods show good agreement for this active region.

### Duration Comparison

This section investigates the difference in sunspot durations between the Walker sample and the SSTrack sample.

**Figure 9:**
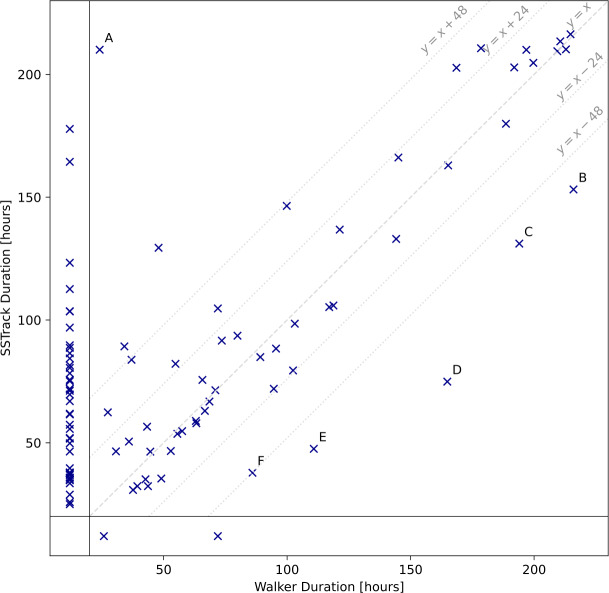
Duration comparison of sunspots identified by the Walker method and the SSTrack method. Sunspots identified only by the SSTrack method are plotted on the left of the figure, and sunspots identified only by Walker are at the bottom of the figure.

Figure 9 shows a scatter plot of the durations of the 54 sunspots identified by both the Walker and SSTrack methods. Generally, the SSTrack method and the Walker method showed good agreement, with the SSTrack method tracking 39 sunspots for ± 24 hours of the Walker duration. The SSTrack method is able to identify sunspots for a longer duration, with 10 of the sunspots being tracked for at least twenty-four hours longer with the SSTrack method over the Walker method. There is one extreme case, AR 11823 (denoted as sunspot A in the plot), where the SSTrack method tracked a sunspot for over 210 hours, whilst the Walker method only tracked the sunspot for 24 hours. There are five sunspots that are tracked using the Walker method for over forty-eight hours more than with the SSTrack method, AR 11793, 11756, 11734, 11755 and 11732 (point B, C, D and E in the plot, respectively). Active region 11756 is discussed further in Section 3.2.1. All sunspots that have more than 48-hours difference in observed duration between the two methods are presented in Table 2.

**Table 2:** Active regions where the Walker method tracked sunspots for significantly different durations than the SSTrack method.

AR	Why
A. 11823	Walker only tracks the sunspot for a 24-hour period, and is due to a mistake in the Walker dataset.
B. 11734	SSTrack identifies sunspot fragmentation, which shortens the observed duration of sunspot $A_{P}$.
C. 11756	SSTrack identifies sunspot fragmentation, which is not recorded in the Walker dataset. This fragmentation reduces the recorded duration of the sunspot in the SSTrack dataset. This sunspot is discussed in Section 3.2.1.
D. 11734	The Walker method identifies sunspot $C_{W}$ as a single structure, whereas the SSTrack method resolves the same region to three smaller sunspots.
E. 11755	SSTrack identifies sunspot fragmentation, whereas the Walker methods does not.
F. 11732	Walker allows analysis of sunspot $B_{W}$ beyond the area criteria cut-off point, whereas SSTrack does not.

#### Analysis of AR 11756: Walker Records a Longer Duration

**Figure 10:**
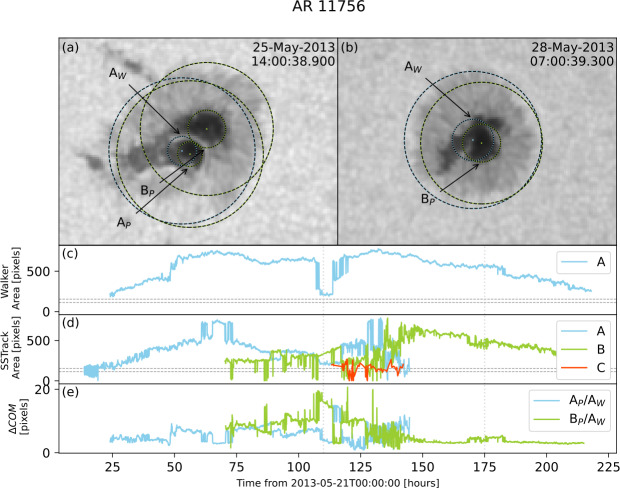
Tracking profiles for AR 11756: (**a,b**) image of the sunspots from 25 May 2013 at 14:00:39 UT and 28 May 2013 at 07:00:39 UT, respectively, with the identified penumbral and umbral radii overlaid from both identification methods; (**c**): the reprojected umbral area profile for the sunspots identified by the Walker method; (**d**): the reprojected umbral area profile for the sunspots identified by the SSTrack method; (**e**): The difference in recorded sunspot centre of mass for the comparable sunspots identified by both methods.

The Walker method tracks sunspot $A_{W}$ for 194 hours, while the SSTrack method only tracks sunspot $A_{P}$ for 131 hours. Figures 10c and 10d show that the SSTrack method identifies sunspot $A_{P}$ 12 hours before the Walker method first identifies the sunspot, but the Walker method continues to track the sunspot for much longer. This active region undergoes a split at 110 hours. The Walker method identifies this structural change but merges the umbral regions back together afterward. The SSTrack method identifies the change in umbral structure slightly earlier, and tracks each component separately ($A_{P}$ and $B_{P}$). Because of this, the full duration of sunspot $A_{W}$ is much longer than the duration of sunspot $A_{P}$, but the remaining component in the SSTrack dataset, sunspot $B_{P}$, is tracked until the final observation of sunspot $A_{W}$, so the combined components $A_{P}$ and $B_{P}$ are tracked for longer than $A_{W}$.

### Rotation Comparison

**Figure 11:**
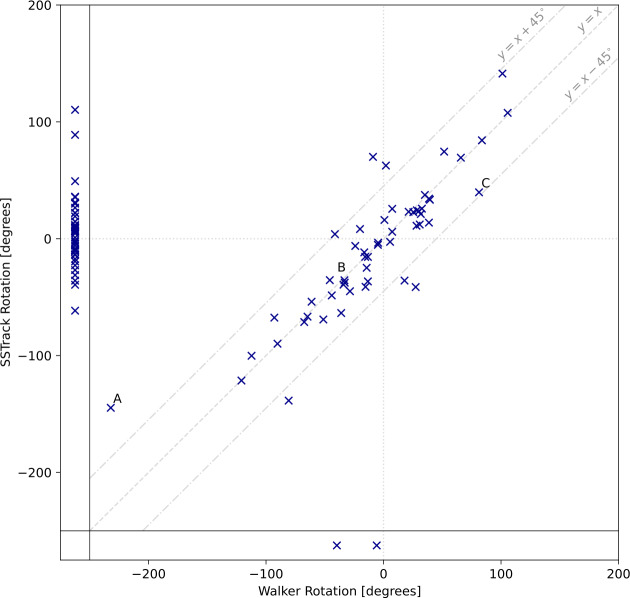
Net rotation comparison (in degrees) of sunspots identified using the Walker method and the SSTrack method. Sunspots only tracked using the SSTrack method are on the left of the figure, and sunspots only tracked using the Walker method are at the bottom of the figure.

**Figure 12:**
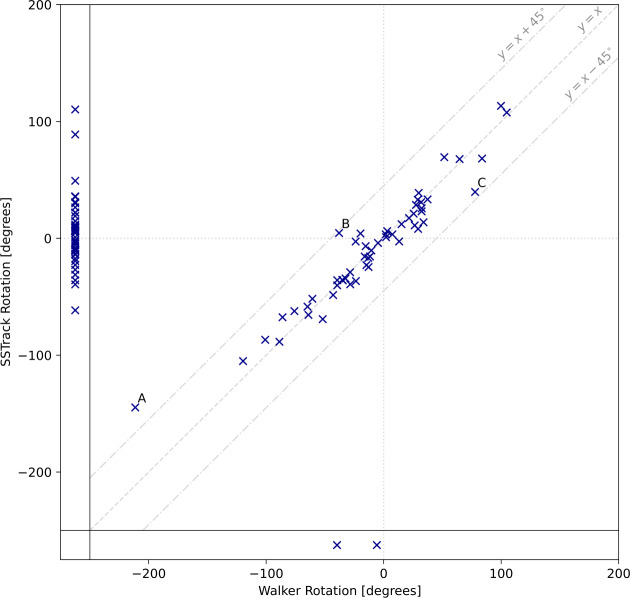
Net rotation comparison (in degrees) of sunspots identified using the Walker method and the SSTrack method using only observations found with both methods. Sunspots only tracked using the SSTrack method are on the left of the figure, and sunspots only tracked using the Walker method are at the bottom of the figure.

The rotation profile of each sunspot about its umbral centre is calculated for both the Walker and SSTrack method. The method to calculate rotations for each sample is given in Brown and Walker (2021) with the difference being that the different centres of mass for each sample are used. The cumulative rotation of each sunspot is shown as a scatter plot in Figure 11. This shows that the majority of commonly identified sunspots in the SSTrack sample have a rotation of ${\pm}\,45$° of the Walker method rotation calculations. This is a relatively large spread. However, Figure 12 shows how the situation improves when only the rotation during the overlapping periods is considered. The sunspots have more similar rotation profiles, with three notable outliers, active region 11762, 11809 and 11793 (A, B and C, respectively). These active regions are discussed in Table 3.

**Table 3:** Active regions where the Walker method measures significantly different sunspot rotation than the SSTrack method.

AR	Why
A. 11762	The sunspot undergoes a period of elongation before fragmenting, and the Walker centre of mass calculations are more strongly influenced by the deformation than the SSTrack calculations. This active region is discussed in Section 3.3.1.
B. 11809	The SSTrack method identifies that sunspot $A_{P}$ undergoes fragmentation, whereas the Walker method does not. Because of this, the Walker penumbral bounds contain some umbral pixels, which could influence the rotation calculations.
C. 11793	The SSTrack identifies sunspot fragmentation that is not picked up by the Walker method, leading to the change in calculated sunspot rotation.

#### Analysis of AR 11762: Increased Walker Rotation Case

**Figure 13:**
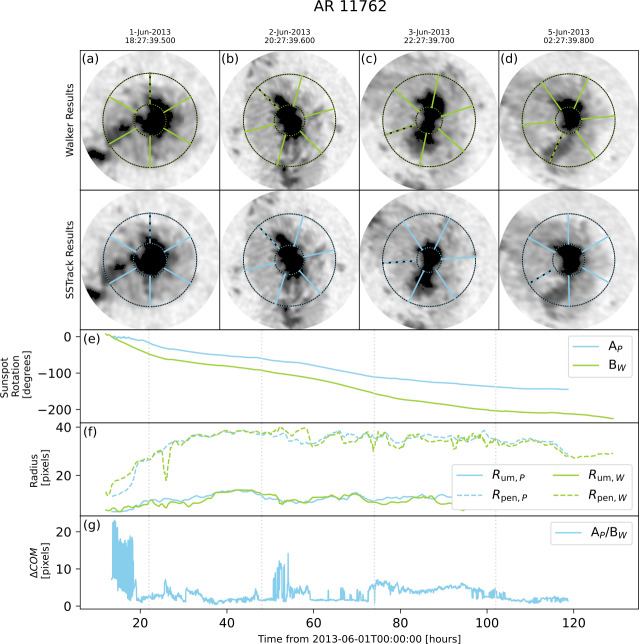
Rotation profiles for AR 11762: (**a, b, c, d**) reprojected image of the target sunspot to the solar centre from 1 June 2013 at 18:27:40 UT, 2 June 2013 at 20:27:40 UT, 3 June 2013 at 22:27:40 and 5 June 2013 at 02:27:40 UT, respectively, with the identified penumbral and umbral radii overlaid and the rotation indicated by the dashed spoke; (**e**) the recorded net rotations of the sunspot in degrees using the Walker and SSTrack samples; (**f**) the penumbral and umbral radii profiles of the sunspot from the Walker and SSTrack samples; (**g**) the difference in recorded sunspot centre of mass for the comparable sunspots identified by both methods.

Point A in Figure 12 comes from active region 11762 which contains a sunspot ($A_{P}$ and $B_{W}$) that is recorded to experience over 45° more rotation using the Walker method than the SSTrack method. Figure 13e shows the greatest variation in rotation occurs at the start of the observation period for both methods, and this difference is also recorded in the change of centre of mass (Figure 13g). This variation also coincides with the period of elongation the sunspot undergoes before eventually fragmenting, the difference is a signature of the Walker calculations being more strongly offset by the fragmentation that is occurring in the lower-left corner of Figure 13a. Subsequent observations have a much more similar rotation pattern.

## Discussion and Conclusions

This article has detailed a new method to automatically identify and track sunspots using data from the Solar Dynamics Observatory’s Helioseismic and Magnetic Imager (SSTrack). The resulting metadata from this method can be fed into various sunspot dynamic analysis methods to investigate the correlation with sunspot dynamics and the build up of energy within active regions. The method is able to identify variations in the umbral structure of a sunspot, such as sunspot splitting and mergers, and track each component accurately.

The method is tested on a four-month sample of data that had been previously analysed using a semi-automatic method by Walker (2018). Three questions are posed;

- is the SSTrack method able to identify all sunspots found by the Walker method
- is the SSTrack method able to track each commonly-found sunspot for the same duration as the Walker method
- how do the rotation profiles of the sunspots compare between methods.

The SSTrack method was able to identify 97 sunspots over 47 active regions, including 54 of the 56 sunspots that were found using the Walker method, with the two unidentified sunspots belonging to active regions 11824 and 11835. The missing sunspot from AR 11824 was identified using the SSTrack method, but the sunspot split into two structures approximately 17 hours after identification. Due to this structural change the sunspot did not meet Walker’s 24-hour size criteria for the rotation analysis, and was not included in the final dataset. The Walker method identified a structural change, but was not designed to handle splitting or merging sunspots, so considered the sunspots to be a single structure for 24.6 hours, so it was included in the final dataset. The Walker method also identified an extra sunspot in AR 11835, however this sunspot is included in the SSTrack dataset. Both methods identify the splitting of sunspot $A_{P}$ and $A_{W}$, however the Walker method records this sunspot after the split as sunspot $AA_{W}$, whilst the SSTrack method records the sunspot as a continuation of $A_{P}$. Due to the difference in naming convention, it appears that the Walker method identifies a sunspot that the SSTrack method does not, however this is not the case. Generally the SSTrack method was able to reliably detect more complex sunspot structures than the Walker method.

When comparing the duration of the commonly identified sunspots, the SSTrack method typically shows good agreement with the Walker method, with 39 sunspots being visible in the SSTrack method for ± 24-hours of the Walker method. The periods observed by both methods had very good agreement, with less than 10 pixels variation between the centre of the sunspots for most observations. There were five cases where Walker was able to track a sunspot for at least forty-eight hours longer than those identified with the SSTrack method. These are contained in AR 11793, 11756, 11734, 11755 and 11732. In each of these cases the SSTrack method was able to detect structural changes within the sunspot under investigation. Each of these sunspots split into multiple, smaller sunspots over the observation period, with some of these components dissipating into the penumbra. Because of the added sensitivity regarding sunspot splits and merges from the SSTrack method, the duration of these splitting sunspots is much less than with the Walker method, which typically ignores this substructure and considers them to be one single sunspot.

The rotation about umbral centres was calculated for each sunspot using the tracking data from both methods. When comparing all observations of the 54 common sunspots, there was a large amount of variation between the methods, with seven sunspots having a difference in rotation greater than 45°. When only comparing the rotation during overlapping observations per sunspot, there was much greater agreement between the two methods, with only one sunspot having more than 45° difference between the methods. The largest discrepancies in the rotation occur when a sunspot undergoes fragmentation. The elongation of the sunspot in the run up to fragmentation has a larger influence on the centre of mass calculations for the Walker method (where SSTrack typically identifies the fragmentation earlier and the weighted centre of mass reduces any impact caused). This can lead to the discrepancies in the measure rotation for each method.

The SSTrack method has a few key advantages over the Walker method. Firstly, the SSTrack method is fully automatic, whereas the Walker method required initial identification and additional parametrisation to be included by hand for each sunspot under analysis. As all sunspots that fulfil the Walker selection criteria are identified without manual interference, the SSTrack method is able to identify 43 more sunspots than the Walker method over this period. The SSTrack method was also able to identify many smaller sunspots, and generally was able to detect sunspots earlier and track them for longer, having knock on effects to the rotation calculations. Secondly, the method is more sensitive to structural changes within the umbral region of a sunspot. The method has successfully tracked the splitting and merging of sunspots throughout the four-month sample, this was a weakness of the Walker method, which successfully tracked the larger scale movements of sunspots but struggled to identify the small-scale structure.

Even in the few cases where the Walker method appears to ‘outperform’ the SSTrack method, further investigation shows this to not be the case, rather the SSTrack method has handled splitting and mergers more elegantly leading to differences in classification rather than a failure to identify sunspots.

The SSTrack algorithm can be used to construct large, robust statistical surveys of sunspot locations and motions over multiple years, potentially an entire solar cycle. This would be at done at a high cadence such that finer-scale evolution, such as sunspot fragmentation and mergers can be detected and monitored. This allows the kinematics of sunspots within active regions to be investigated, for example, the information from SSTrack can be used by algorithms that measure sunspot rotations about their umbral centres (e.g., Brown and Walker 2021) and the tracking information can be used to determine the corotation of sunspots about each other. Furthermore, eruptive behaviour such as solar flares are routinely linked to their source active regions in solar catalogues and studies (e.g., Hernandez Camero, Green, and Piñel Neparidze 2025) have developed automated algorithms to allow this to be carried out with CMEs (where they originate from an active region).

This enables large statistical studies of how active region kinematics influence solar eruptions to be carried out. For example, Walker (2018) develops a method to estimate the energy built-up in an active region by assuming that each sunspot is circular (the cross-section of a cylindrical flux tube) and using the average sunspot area to estimate the average magnetic flux of the active region, this can be used to calculate the added self-helicity for a given amount of rotation, which is then converted into magnetic energy. Walker (2018) uses different ways to combine energies from sunspots to compare the built-up active region energy to the estimated energy released by solar flares over the same period to investigate whether sunspot rotation can provide the energy budget for solar eruptions. A statistical sample produced by SSTrack can be used to carry out this study with less bias, as well as expanding it to include energy input from other kinematics (such as sunspot corotation) and other energy outputs (such as coronal mass ejections).

**Figure 14:**
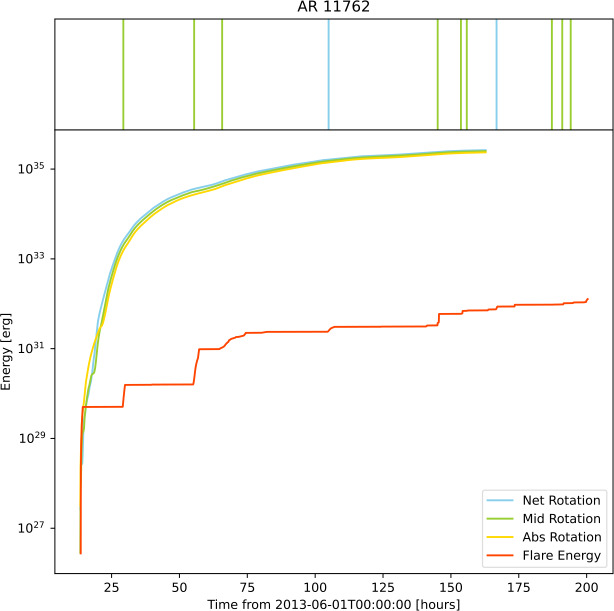
The top figure shows the GOES peak time of all flares associated with active region 11762. Green lines represent C-Class flares, and blue lines represent M-Class flares. There were no X-Class flares associated with this active region. The bottom figure shows the active region rotation energy and flaring energy profiles for AR 11762. The red line represents the radiated flare energy, the blue line represents the net rotation energy, the green line represents the mid rotation energy and the yellow line represents the absolute rotation energy.

This analysis can be refined further to consider the temporal progression of energy input and output to determine whether energy from, e.g., sunspot rotation is being injected into the corona in a timely manner, by calculating the magnetic flux (based on sunspot area) using an observation-by-observation approach instead. Figure 14 shows the cumulative active region energy contribution from the net, mid and absolute rotation profiles, alongside the cumulative bolometric flaring energy for active region 11762. To estimate the cumulative bolometric flaring energy, the GOES time series for each flare associated with the active region is integrated to calculate the total optically thin energy loss. This value is then multiplied by a constant to give the bolometric energy loss. From the figure, the flaring energy is initially similar to the rotation energy. The rotation energy exceeds the flaring energy at 15 hours and from this point on the active region rotation generates more than enough energy to account for the energy released by the solar flares. In this case, sunspot rotation injects sufficient energy in a timely manner for the solar flares, but a statistical study is required to determine whether this is more generally true.

Large statistical studies comparing sunspot kinematics with solar activity will play an important role in clarifying how solar activity is powered and triggered, and developing methods such as SSTrack to create large statistical samples is an important objective.

### Open Data

The rotation profiles generated for the test active regions in this article are available for further scientific use. FITS files for each active region, along with documentation, can be found under DOI 10.17030/uclan.data.00000652.

## Figures and Tables

**Figure 15:**
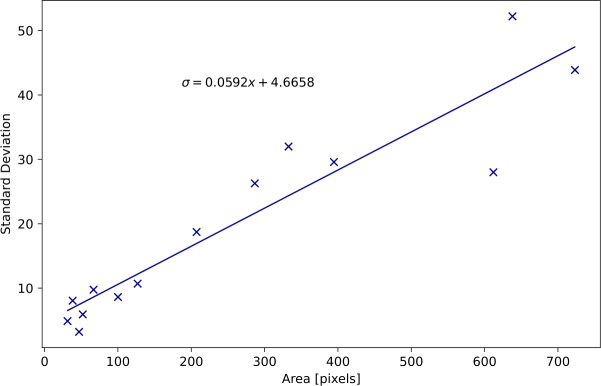
Median area of the 14 sunspots in the sample against their corresponding standard deviation, $s_{i}$. The regression line between these variables is plotted.

## Data Availability

The rotation profiles generated for the test active regions in this article are available for further scientific use. FITS files for each active region, along with documentation, can be found under DOI 10.17030/uclan.data.00000652, URL: https://data.lancashire.ac.uk/id/eprint/652.

## Appendix A: Area Limit Calculation

In order to determine the area limit factor, a small sample of 14 sunspots whose areas range between $39\pi -350\pi~\mathrm{pixel}^{2}$ were selected and their variation in area analysed. The 14 sunspots were chosen because they had a stable structure with little variation in size and did not exhibit any splitting or merging. These are the only sunspots in the four-month sample that meet this criteria.

A median filter, with a 41 observation kernel, is applied to the area profile of each sunspot. The variance of the sunspot area from the running median area is calculated as follows: suppose $A_{i,j}$ is the $j$’th observation for sunspot $i$ and $\tilde{A}_{i,j}$ is the corresponding running median for the area, then the variance is taken as

$$ s_{i}^{2} = \frac{1}{n_{i}-1}\sum _{j=1}^{n_{i}} \left (A_{i,j}- \tilde{A}_{i,j}\right )^{2}, $$

where $n_{i}$ is the number of observations analysed for sunspot $i$.

A median filter is preferred as occasional rogue area measurement can lead to extreme outliers that can bias the mean, where the median filter is unaffected by these outliers.

To determine a relationship between sunspot area and variation in area, a scatter plot is produced and the linear regression line for the data is fit. This can be seen in Figure 15. The best fit regression line is given by

$$ \sigma =0.0592 \times A_{\mathrm{area}} + 4.6658. $$

where $\sigma $ is the area limit factor corresponding to a given sunspot area, $A_{area}$.

## Appendix B: Removed Walker Sunspots

The following sunspots are removed from the Walker sample due to not meeting the area criteria: $B_{W}$ from AR 11748, $B_{W}$ from AR 11765, $B_{W}$ from AR 11772, $B_{W}$ from AR 11800, and $A_{W}$ from AR 11827.

## Appendix C: Rotation File Definition

The data available in the open data rotation FITS profiles includes the sunspots tracked per active region that pass the duration and area filters outlined in Section 2.3. Each sunspot includes the metadata listed in Table 4 and the time series for the quantities listed in Table 5.

**Table 4:** An overview of header data available in the RSS FITS rotation profiles.

Keyword	Description
AR	Active Region number
SPOTNO	Sunspot number in active region (e.g. A, B, C, …)
T0	Reference Time (a numerical time in hours from the reference time is provided in the data fields)
RADEXT1	Minimum annuli radius used during rotation calculation
RADEXT2	Maximum annuli radius used during rotation calculation
THRESH	Umbral threshold applied during rotation calculation
LTHRESH	Lower threshold applied (to ignore dropped data, etc.)
PTHRESH	Penumbnral threshold applied during rotation calculation
MAXDT	Maximum time between observations for tracking (hour)

**Table 5:** An overview of data fields available in the RSS FITS rotation profiles.

Field	Description
FILE	Filename of HMI observation used
TIME	Date/time stamp of observation analysed
DTIME	Time elapsed (in hours) from reference time (T0)
PCENX	Calculated x-coordinate (in pixels) of the centre of the sunspot in the observation
PCENY	Calculated y-coordinate (in pixels) of the centre of the sunspot in the observation
SIGPCX	Calculated one-sigma error on PCENX
SIGPCY	Calculated one-sigma error on PCENY
ACENX	Calculated x-coordinate (in arcsecs) of the centre of the sunspot in the observation
ACENY	Calculated y-coordinate (in arcsecs) of the centre of the sunspot in the observation
UMBRAREA	Calculated reprojected area (i.e., area if the sunspot was at disk centre) of the umbra of the sunspot (pixels with intensity below THRESH)
PANN1	Inner radius (pixels) of the penumbral annulus averages over a 1-hour window centred on the observation
PANN2	Outer radius (pixels) of the penumbral annulus averages over a 1-hour window centred on the observation
SIGPANN1	One-sigma error on PANN1
SIGPANN2	One-sigma error on PANN2
DROTN	Calculated rotation (degrees) between previous observation and current
SIGDROTN	One-sigma error on DROTN
ANGVEL	Bulk angular velocity based on a 1-hour window centred on the observation
SIGAV	One-sigma error on ANGVEL
ROTN	Cumulative rotation of the sunspot from the first observation
SIGR	Total error on ROTN
SIGRM	Error on ROTN due to the method (e.g., error propagation and scale errors)
SIGRP	Error on ROTN due to the error on the penumbral radii
SIGRC	Error on ROTN due to the error on the centre of the sunspot
ABSROTN	Cumulative absolute rotation of the sunspot from the first observation
